# Zinc Favors Triple-Negative Breast Cancer’s Microenvironment Modulation and Cell Plasticity

**DOI:** 10.3390/ijms22179188

**Published:** 2021-08-25

**Authors:** Marina Vogel-González, Dunia Musa-Afaneh, Pilar Rivera Gil, Rubén Vicente

**Affiliations:** 1 Laboratory of Molecular Physiology, Department of Experimental and Health Sciences, Universitat Pompeu Fabra, 08003 Barcelona, Spain; marina.vogel@upf.edu (M.V.-G.); dunia.mf17@gmail.com (D.M.-A.); 2 Integrative Biomedical Materials and Nanomedicine Lab, Department of Experimental and Health Sciences, Pompeu Fabra University, 08003 Barcelona, Spain; pilar.rivera@upf.edu

**Keywords:** zinc, triple-negative breast cancer, metastasis, Zip4

## Abstract

Triple-negative breast cancer (TNBC) tends to metastasize to the brain, a step that worsens the patient’s prognosis. The specific hallmarks that determine successful metastasis are motility and invasion, microenvironment modulation, plasticity, and colonization. Zinc, an essential trace element, has been shown to be involved in all of these processes. In this work, we focus our attention on the potential role of zinc during TNBC metastasis. We used MDA-MB-BrM2 (BrM2) cells, a brain metastasis model derived from the parental TNBC cell line MDA-MB-231. Our studies show that BrM2 cells had double the zinc content of MDA-MB-231 cells. Moreover, exploring different metastatic hallmarks, we found that the zinc concentration is especially important in the microenvironment modulation of brain metastatic cells, enhancing the expression of SerpinB2. Furthermore, we show that zinc promotes the tumorigenic capacity of breast cancer stem cells. In addition, by causing a disturbance in MDA-MB-231 zinc homeostasis by overexpressing the Zip4 transporter, we were able to increase tumorigenicity. Nevertheless, this strategy did not completely recapitulate the BrM2 metastatic phenotype. Altogether, our work suggests that zinc plays an important role in the transformative steps that tumoral cells take to acquire tumorigenic potential and niche specificity.

## 1. Introduction

Triple-negative breast cancer (TNBC) accounts for almost 20% of breast cancers and tends to be more aggressive than non-TNBCs. TNBC has an increased risk of early metastasis and lower 5-year survival than other breast cancers [1]. Given that TNBC lacks expression of the estrogen receptor (ER), progesterone receptor (PR), and human epidermal growth factor receptor 2 (HER2), it cannot be targeted by specific treatments. For this reason, TNBC tumors have a higher rate of distant recurrence [2]. Breast cancer is the second leading cause of brain metastasis, with TNBC responsible for around 40% of cases [3], and the worst prognosis, with an overall survival of 4.9 months [4]. 

Metastasis is considered the ultimate manifestation of cancer. Cells, after acquiring all of the characteristics needed to be tumorous, present the so-called hallmarks of metastasis: motility and invasion, microenvironment modulation, plasticity, and colonization [5]. The motility and invasion step is essential for cells to arrive at the secondary niche, where metastasis is developed. Once there, it is crucial for the cells to modulate the microenvironment so they can survive and progress in their new niche [6]. In brain metastasis, overexpression and secretion by cancer cells of a molecule called SerpinB2 has been reported to play a key role in tumor escape from brain defenses [7]. Tumor plasticity is also needed to generate the heterogeneity responsible for survival and progression. Cancer stem cells (CSCs) provide this plasticity. CSCs are a small population of dormant, de-differentiated cancer cells with the capacity of self-renewal that can re-establish a heterogeneous population of tumor cells, guaranteeing tumor diversity [8]. In addition, CSCs are resistant to therapy and their presence leads to relapse [9], which are known characteristics of TNBC. Finally, to achieve colonization of the secondary niche, the tumor has to proliferate in situ, thanks to the interaction between the microenvironment and cells derived from these CSCs [5].

Zinc is the second most abundant trace element in the human body. Its homeostasis is essential, and it plays key roles as a structural, catalytic, and signaling element [10]. In this way, zinc is involved in processes, such as proliferation [11,12,13], migration [14,15,16], differentiation [17,18,19], and survival [20], in different cell types. Zinc dysregulation has been reported to appear in several cancers, in both serum concentration and tumor content [21]. This scenario is caused by alterations in the most important proteins that maintain zinc homeostasis: zinc transporters. Thus, modification of the physiological expression of certain Zips and some ZnT transporters has been linked to the progression of different kinds of cancers, such as Zip1 downregulation in prostatic cancer and Zip4 upregulation in pancreatic cancer [21].

In breast cancer, it has been reported that patients have a reduced zinc concentration in the serum and an abnormally elevated zinc content in tumors [22]. In addition, zinc concentration correlates with the histological malignancy grade, thus it has been proposed as a biomarker for breast cancer [23]. An imbalance in the expression of some Zips has been proven to be involved in different ways with the progression and aggressiveness of this condition. Zip6 appears to be downregulated in high-grade primary estrogen-receptor-positive tumors [24]. Zip10 has been associated with invasion and metastasis [25]. Both Zip6 and Zip10 increase cell migration and help with the epithelial–mesenchymal transition (EMT) [16,25]. Zip7 has also been shown to be high in tamoxifen-resistant estrogen-receptor-positive breast cancer [26].

Although it is now well established that zinc imbalance is involved in the tumorous process in many cancers, including breast cancer, studying its role in specific models is still crucial due to the heterogeneity of this condition. In this context, our work focuses on the role of zinc homeostasis and the consequences of zinc supplementation in TNBC metastasis. For this purpose, we used MDA-MB-231 cells as a model of TNBC and MDA-MB-231-BrM2 cells (BrM2) as a model of TNBC specifically metastasized to the brain.

We characterized the impact of zinc content on the different hallmarks of metastasis in both cell lines. Our results show that brain metastatic breast cancer cells acquire an abnormally high zinc concentration. This phenomenon favors CSCs tumorigenicity and microenvironment modulation of their new niche.

## 2. Results

### 2.1. Characterization of Cellular Zinc Content in TNBC Cell Lines

**Figure 1:**
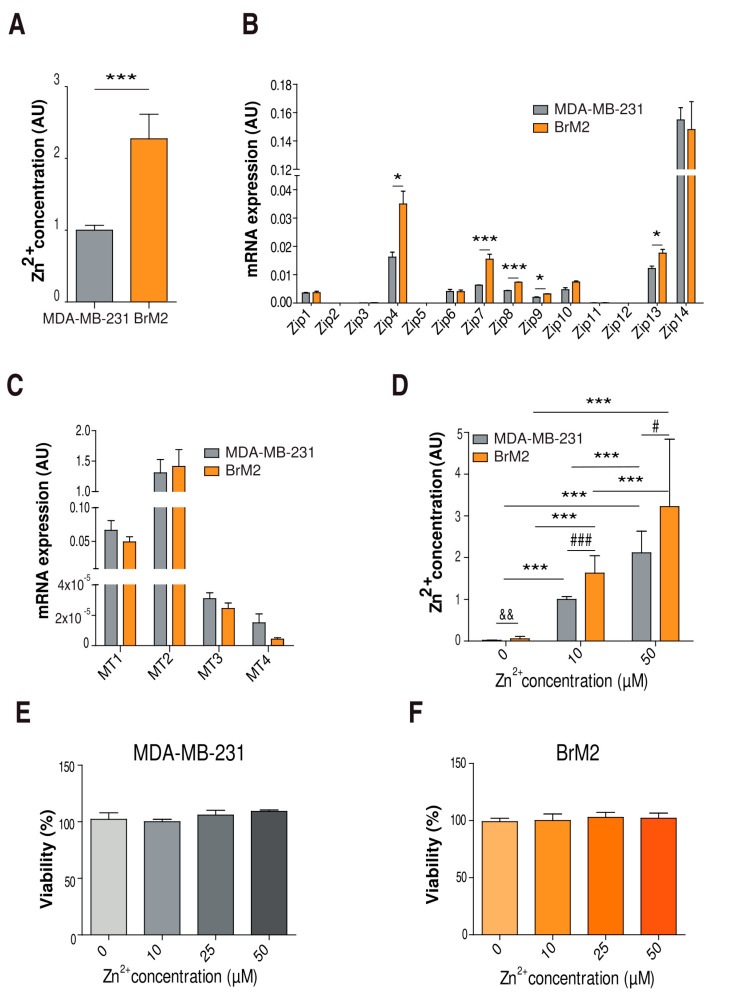
Characterization of zinc homeostasis of MDA-MB-231 and BrM2 cells. (**A**) Evaluation of zinc content by flow cytometry using Zinquin in basal conditions (*n* = 12); *** *p* < 0.005 using Mann–Whitney test. (**B**,**C**) Real-time PCR comparing expression of Zip transporter family (*n* = 3) and metallothioneins (*n* = 6–9) in MDA-MB-231 and BrM2 cells in basal conditions. 2-DCT plotted using GAPDH as a housekeeping gene; * *p* < 0.05, *** *p* < 0.005 using *t*-test. (**D**) Evaluation of zinc content by flow cytometry using Zinquin after 24 h of treatment with 0, 10, and 50 µM of ZnSO_4_ (*n* = 6–9); *** *p* < 0.005 using ANOVA and Tukey’s multiple comparison test; && *p* < 0.01 using Mann–Whitney test; # *p* < 0.05, ### *p* < 0.005 using *t*-test. (**E**,**F**) Evaluation of cell viability by MTT in MDA-MB-231 and BrM2 cells grown at different zinc concentrations for 48 h (*n* = 3).

In order to delineate the relevance of intracellular zinc in TNBC brain metastasis, first we compared the zinc concentration in the basal condition between MDA-MB-231 and BrM2 cells, our TNBC model for brain metastasis. Using Zinquin as a zinc content reporter, we observed that BrM2 cells had double the zinc content of MDA-MB-231 cells (Figure 1A). We then characterized the expression of several zinc homeostasis molecular players, such as Zip transporters and metallothioneins. Our mRNA expression analysis showed upregulation of Zip4, Zip7, Zip8, Zip9, and Zip13 expression in the BrM2 cells compared to the MDA-MB-231 cells (Figure 1B). Regarding the metallothioneins, no significant changes were observed (Figure 1C). Altogether, our results show an enrichment of zinc content and altered zinc homeostasis in the brain metastatic BrM2 lineage.

Then, we set up our experimental conditions with different extracellular zinc concentrations in order to study how zinc might modulate metastatic and niche-specific characteristics. We treated both cell lines with 0, 10, and 50 µM ZnSO_4_ to reproduce zinc deficiency, physiological zinc, and zinc supplementation, respectively. After 24 h, we observed significant differences in all conditions (Figure 1D). After 48 h under these conditions, our MTT assays showed no alterations in cell viability (Figure 1E,F).

### 2.2. TNBC Zinc Modulation in Cell Migration, Motility, and Invasion

**Figure 2:**
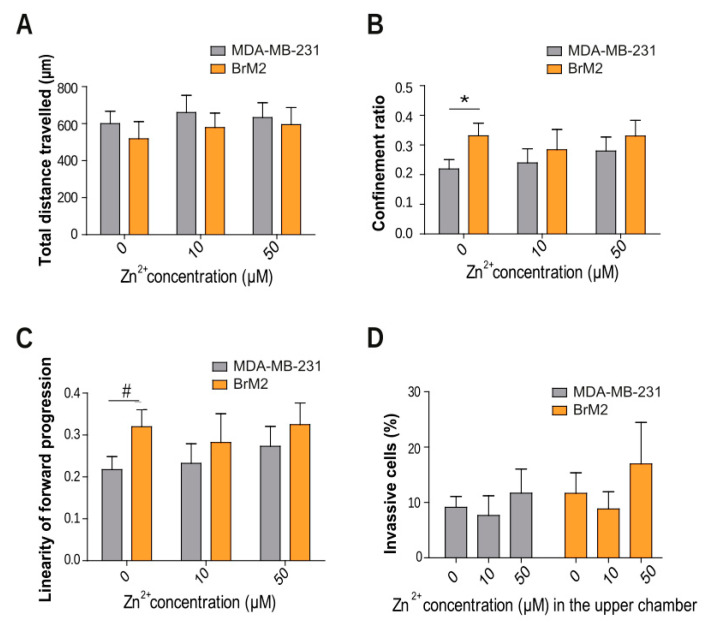
Characterization of migration, motility, and invasion of MDA-MB-231 and BrM2 cells in different zinc concentrations. Comparison of representative cell migration parameters, including (**A**) total distance travelled, (**B**) confinement ratio (net distance/total distance travelled), and (**C**) linearity of forward progression ((net distance/total track time)/mean speed) (*n* = 17–26); * *p* < 0.05 using *t*-test, # *p* < 0.05 using Mann–Whitney test. (**D**) Percentage of cells, incubated at different zinc concentrations, that invaded the bottom chamber, with normal media (*n* = 4).

Cell migration, an essential aspect of metastasis [5], is known to be affected by zinc signaling through different transporters [14,15,16]. We recorded random migration of our TNBC cell lines for 24 h in different zinc concentrations. Both cell lines, independent of the zinc concentration, showed no differences in the majority of measured parameters: total distance travelled (Figure 2A), mean velocity, max distance travelled, and mean straight line seed (data not shown). Our data also reveal a similar pattern of migration in both MDA-MB-231 and BrM2 cells. However, in the absence of zinc, two parameters were significantly lower in the MDA-MB-231 cells than the BrM2 cells: the confinement ratio (Figure 2B) and the linearity of forward progression (Figure 2C). These measure the efficiency of displacement from the initial position and how linear the progression is, respectively. This difference was not observed in the presence of zinc. Moreover, we studied whether zinc might influence the cell invasion capacity. Thus, TNBC cells were seeded in transwells covered with a physiological matrix and cultured for 72 h. Our results showed no significant differences between cell types or zinc concentrations (Figure 2D).

### 2.3. Implication of Zinc Homeostasis in TNBC Brain Microenvironment Modulation

**Figure 3:**
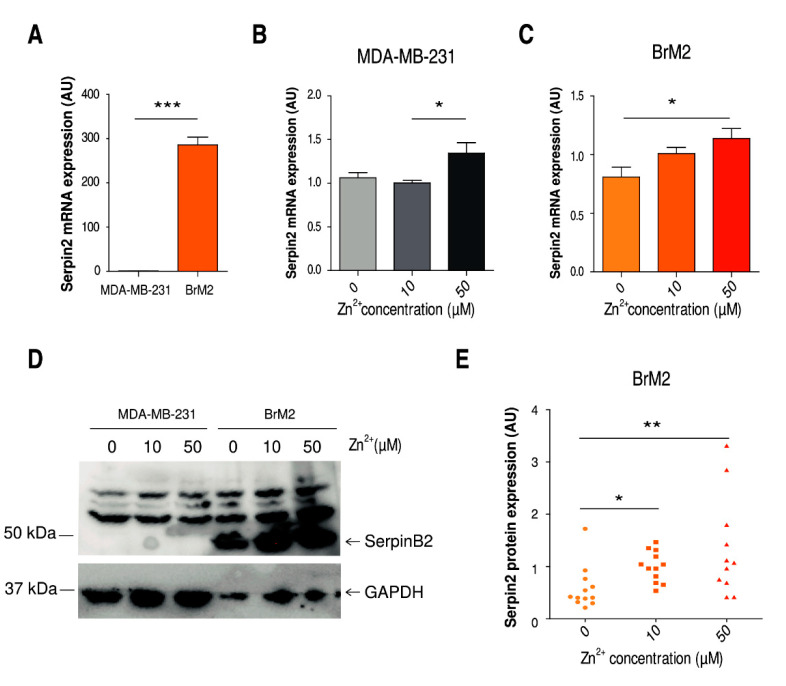
Characterization of SerpinB2 expression at the mRNA and protein level in MDA-MB-231 and BrM2 cells (**A**) RT-PCR comparing SerpinB2 expression in MDA-MB-231 and BrM2 cells in basal conditions. 2-DDCT plotted using GAPDH as the housekeeping gene and MDA-MB-231 as the control line (*n* = 9); *** *p* < 0.001 using t-test. (**B**,**C**) RT-PCR comparing SerpinB2 in 0, 10, and 50 µM of ZnSO_4_ after 24 h of treatment. 2-DDCT plotted using GAPDH as the housekeeping gene and normalized to the 10 µM condition of each cell line (*n* = 6); * *p* < 0.05 using ANOVA and Tukey’s multiple comparison test. (**D**) Representative Western blot against SerpinB2 and GAPDH in MDA-MB-231 and BrM2 after 24 h of treatment with 0, 10, and 50 µM of ZnSO_4_. (**E**) Quantification of SerpinB2 protein expression normalized by GAPDH protein expression in BrM2 cells after 24 h of treatment with 0, 10, and 50 µM of ZnSO_4_ (*n* = 11–12); * *p* < 0.05, ** *p* < 0.01 using Dunn’s multiple comparison test.

Metastatic cells in the brain must confront highly complex tissue and escape from local defenses. In this context, the secretion of the protease SerpinB2 by breast cancer cells has been reported to be a key mechanism modulating the brain microenvironment [7]. As previously described [27,28], BrM2 cells showed increased expression of SerpinB2 at the mRNA and protein level compared to MDA-MB-231 cells (Figure 3A). We decided to further explore whether the cellular zinc content might contribute to SerpinB2 expression. Both cell lines showed a rise in the mRNA expression levels of SerpinB2 when with zinc supplementation (Figure 3B,C). However, independent of the cellular zinc content, SerpinB2 was not detectable by Western blot in the MDA-MB-231 cells (Figure 3D). In the BrM2 cells, on the other hand, we observed that the expression of this protease was strongly induced by cellular zinc concentration (Figure 3D,E). In addition, we studied this regulation in the lung metastatic MDA-MB-231-LM2 cell line (LM2). Despite this cell line presenting lower SerpinB2 expression levels than BrM2 cells, we observed a zinc dependent regulation of SerpinB2 as well (Supplementary Materials Figure S1).

### 2.4. Role of Zinc in Cell Proliferation

**Figure 4:**
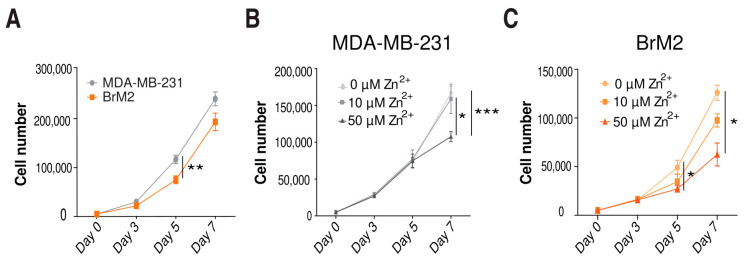
Proliferation assays in basal conditions and with changed zinc concentration. (**A**) Proliferation analysis comparing MDA-MB-231 and BrM2 cells. Cells were counted on days 3, 5, and 7 (*n* = 15–18); ** *p* < 0.01 using t-test. (**B**) Proliferation analysis comparing MDA-MB-231 cells growing in 0, 10, and 50 µM of ZnSO_4_. Cells were counted on days 3, 5, and 7 (*n* = 9–15); * *p* < 0.05, *** *p* < 0.001 using *t*-test. (**C**) Proliferation analysis comparing BrM2 cells growing in 0, 10, and 50 µM of ZnSO_4_. Cells were counted on days 3, 5, and 7 (*n* = 6); * *p* < 0.05 using *t*-test.

The success of metastasis involves cell proliferation and colonization of the new niche [5]. Zinc and zinc transporters have been reported to modulate cell proliferation in several cell lines [11,12,13,29]. In our TNBC model, we found a lower proliferation rate in BrM2 cells compared with MDA-MB-231 cells (Figure 4A). Considering the differences in zinc homeostasis between cell lines (Figure 1A), we studied the effect that zinc content might have on proliferation. Consistently, with zinc supplementation, the proliferation rate of MDA-MB-231 and BrM2 cells decreased compared to the condition with no zinc (Figure 4B,C).

### 2.5. Influence of Zinc on CSC Tumorigenicity

**Figure 5:**
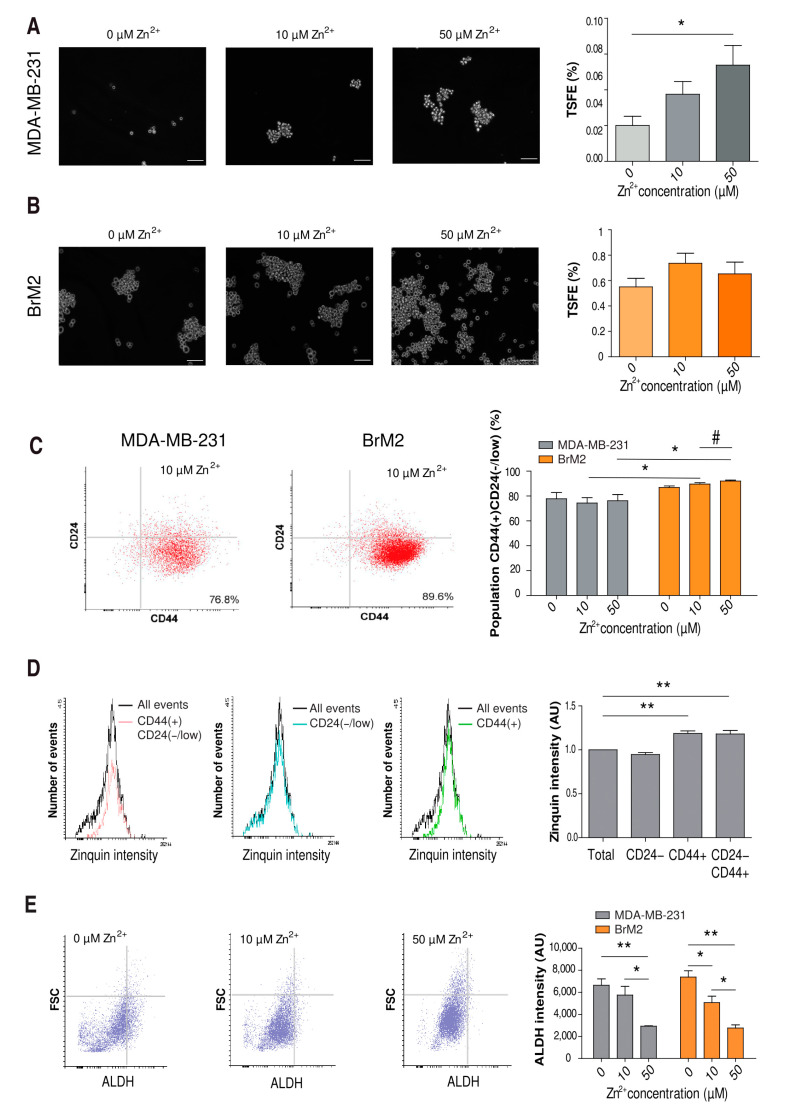
Characterization of tumorspheres in different zinc concentrations. (**A**,**B**) Left, representative pictures of MDA-MB-231 (**A**) and BrM2 (**B**) tumorspheres formed at 0, 10, and 50 µM of ZnSO_4_ after 21 days of differentiation. Scale bar = 100 µm. Right, bar graph showing mean of TSFE for each condition. MDA-MB-231 cells (*n* = 24) * *p* < 0.05 using Dunn’s multiple comparison test. BrM2 cells (*n* = 22–27). (**C**) Representative flow cytometry dot blots of MDA-MB-231 and BrM2 tumorspheres stained with CD24-PE and CD44-APC. Right, percentage of CD44(+)CD24(−/low) cells after generating tumorspheres (*n* = 4–6) * *p* < 0.05 using *t*-test, # *p* < 0.05 using ANOVA and Tukey’s multiple comparison test. (**D**) Evaluation of zinc content with Zinquin by flow cytometry in MDA-MB-231 cells showing total events and (left to right) CD44(+)CD24(−/low), CD24(−/low), and CD44(+) populations. Representative histograms: right, analysis of these populations normalized by the Zinquin intensity of the total cell population from each experiment (*n* = 3) ** *p* < 0.01 using Dunnett’s multiple comparison. (**E**) Representative flow cytometry dot blots evaluating ALDH expression in MDA-MB-231 at 0, 10, and 50 µM of ZnSO_4_ (left). Analysis of ALDH expression in MDA-MB-231 and BrM2 at 0, 10, and 50 µM of ZnSO_4_ (*n* = 3) * *p* < 0.05, ** *p* < 0.01 using ANOVA and Tukey’s multiple comparison test.

Low proliferation is a common feature of dormant CSCs [30]. These cells, and specifically in TNBC [31], are essential for the success of metastasis due to their capacity for generating a whole new tumor with the needed heterogeneity [8]. Interestingly, SerpinB2 has been proposed as an indicator of tumorigenicity [32]. Therefore, we wondered whether zinc exposure could enhance the population of CSCs and their tumorigenicity in our model. It has been established that stem cell-like properties are enriched in non-adhesive sphere culture conditions, in which cells form tumorspheres [32]. We used this system and manipulated the zinc concentrations to be 0, 10, and 50 µM. In all conditions, tumorsphere formation efficiency (TSFE) was higher in BrM2 cells than MDA-MB-231 cells (Figure 5A,B). Moreover, our experiments showed that in MDA-MB-231 cells, TSFE was positively dependent on the presence of zinc in the growth medium (Figure 5A). In BrM2 cells, TSFE numbers were around 10-fold higher than in MDA-MB-231 cells and did not show significant differences between zinc concentrations (Figure 5B).

In parallel, we characterized the cellular composition of the tumorspheres obtained by flow cytometry. Breast CSCs are known to be CD44(+)CD24(−/low) [31]. When comparing the two cell lines for each zinc concentration, we found that the CSC population was significantly higher in the BrM2 than the MDA-MB-231 cells at 10 and 50 µM zinc concentrations (Figure 5C). No major differences in CSC content were found when modulating the zinc concentration, suggesting that zinc promotes the ability of CSCs to form tumorspheres but does not influence their final composition (Figure 5C). In addition, we found a correlation between cellular zinc content and CSC features in tumorspheres (Figure 5D). Thus, the histogram of Zinquin fluorescence intensity in MDA-MB-231 cells shifted toward higher intensity values in CD44(+)CD24(−/low) cells compared to total cells. This correlation was maintained when comparing CD44(+) cells but not CD24(−/low) cells alone. In addition, we characterized the expression of aldehyde dehydrogenase (ALDH), an epithelial-like state marker in breast CSCs, of the tumorspheres [33]. We did not observe major differences in ALDH fluorescence between MDA-MB-231 and BrM2 cells. However, our results showed a strong reduction in ALDH fluorescence depending on the zinc concentration in both cell lines (Figure 5E).

We wanted to further study stemness in our cell model. Therefore, we examined whether there was a correlation between zinc content and the expression levels of the well-known stem-cell markers Oct4, Sox2, and Nanog [34], as well as other zinc-dependent tumorigenic markers, such as ZEB1 [35] and SerpinB2 [32]. Even though we observed some interesting tendencies in MDA-MB-231 cells, no significant differences were obtained for any marker (Supplementary Materials Figure S2).

### 2.6. Influence of Zinc on CSC Tumorigenicity

**Figure 6:**
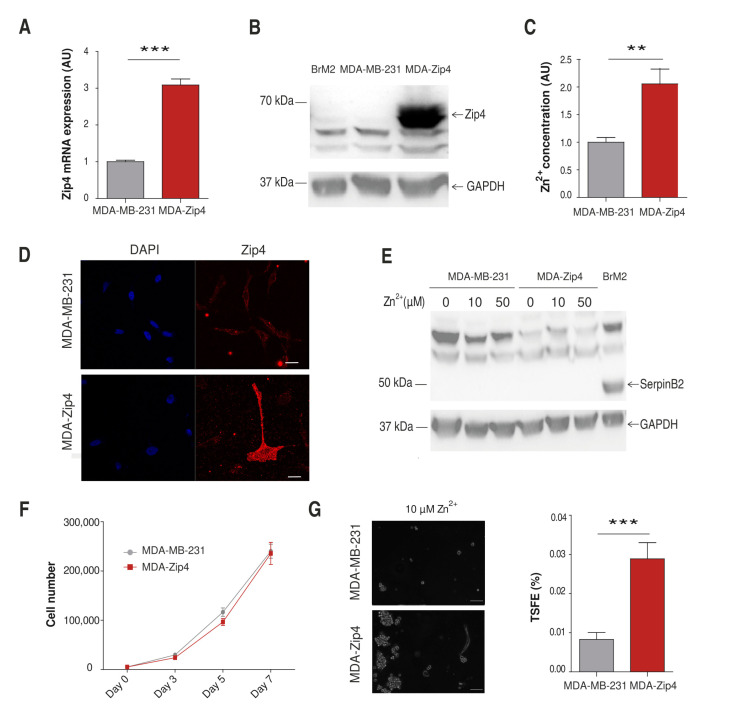
Characterization of the MDA-Zip4 cell line and Zip4 overexpression in the brain metastatic process. (**A**) RT-PCR comparing SerpinB2 expression in MDA-MB-231 and MDA-Zip4 cells in basal conditions. 2-DCT plotted using GAPDH as the housekeeping gene (*n* = 4); *** *p* < 0.005 using *t*-test. (**B**) Representative Western blot against Zip4 and GAPDH in MDA-MB-231, MDA-Zip4, and BrM2 cells. (**C**) Evaluation of zinc content by flow cytometry using Zinquin in 10 µM of ZnSO_4_ (*n* = 9); ** *p* < 0.01 using *t*-test. (**D**) Representative pictures of MDA-MB-231 and MDA-Zip4 DAPI (blue) and Zip4 expression (red). Scale bar = 20 µm. (**E**) Representative Western blot against SerpinB2 and GAPDH after 24 of treatment with 0, 10, and 50 µM of ZnSO_4_ in MDA-MB-231 and MDA-Zip4, and in BrM2 cells in basal conditions. (**F**) Proliferation analysis comparing MDA-MB-231 and MDA-Zip4 cells. Cells were counted on days 3, 5, and 7 (*n* = 12–15). (**G**) Left, representative pictures of MDA-MB-231 (top) and MDA-Zip4 (bottom) tumorspheres formed with 10 µM of ZnSO_4_ after 21 days of differentiation. Scale bar = 100 µm. Right, bar graph showing mean of TSFE for MDA-MB-231 and MDA-Zip4 cells (*n* = 35–36); *** *p* < 0.001 using Mann–Whitney’s *t*-test.

Our results comparing the two TNBC models reveal that zinc induces some malignancy traits of brain metastatic cancer cells. In order to determine the specific molecular players involved in this process, we focused our attention on Zip4, a transporter differentially expressed between lines (Figure 1B) that has been previously linked to tumor progression. In pancreatic cancer, Zip4 overexpression involves increased cell proliferation, survival, migration, and invasion [36,37,38]. Moreover, according to the cBioPortal for Cancer Genomics [39,40], 20% of breast cancer patients have an amplification in the Zip4 coding gene, Slc39a4. Importantly, these patients have reduced survival compared with others [39,40] (Supplementary Materials Figure S3). Therefore, we generated an MDA-MB-231 cell line with Zip4 constitutive overexpression (MDA-Zip4). We confirmed the upregulation of Zip4 in the MDA-Zip4 cells by qPCR and Western blot (Figure 6A,B). Our zinc content measurements using Zinquin showed an increased cytosolic zinc concentration in MDA-Zip4 cells compared to MDA-MB-231 cells (Figure 6C). We confirmed that Zip4 was correctly expressed at the plasma membrane (Figure 6D). Then, we explored whether Zip4 overexpression was sufficient to promote SerpinB2. However, similar to MDA-MB-231 cells, SerpinB2 protein was not detectable in MDA-Zip4 cells at any zinc concentration studied (Figure 6E). Regarding cell proliferation, we saw no difference between cell lines (Figure 6F). On the contrary, our studies show that Zip4 affected the tumorsphere generation of MDA-MB-231 cells. We observed that MDA-Zip4 cells formed more tumorspheres than MDA-MDA-231 control cells (Figure 6G).

## 3. Discussion

In the fight against cancer, we aim to find novel treatments by identifying unique molecular features of each tumor subtype and understand the different tumorigenesis pathways. TNBC contributes to 40% of brain metastasis coming from the breast [3] in a very quick process, against which there are no specific tools [2]. In this work, we used BrM2 cells, a brain metastasis model obtained by seeding MDA-MB-231 cells into mice twice and re-collecting those that specifically establish a secondary tumor in the brain [27]. After two brain metastases, BrM2 cells undergo a highly selective process that confers a specific phenotype. As expected, our work shows that BrM2 cells have metastatic capacity to successfully colonize the brain compared to the parental line. Interestingly, BrM2 cells showed a higher zinc content than the MDA-MB-231 parental line. The results obtained from this study highlight the impact of the cellular zinc content on specific hallmarks of brain metastasis.

An initial step during metastasis is the emancipation of cells from the primary tumor, increasing their motility, migration, and invasion [5]. Zinc was previously shown to be involved in cell migration in breast cancer cells [16,41,42,43]. In our TNBC model, MDA-MB-231 cells showed dependency on zinc for the efficiency and linearity of displacement, which was absent in BrM2 cells. However, we saw no major alteration in the invasion capacity using transwells when altering the zinc concentration in TNBC cells. Altogether, our data show a milder phenotype in migration and invasion depending on extracellular zinc compared to the results obtained by others using the zinc chelator TPEN or knocking down Zip10 [41]. Our experimental conditions probably had a milder impact on zinc cellular homeostasis. 

SerpinB2 is an inhibitor of the urokinase plasminogen activator, which promotes cancer cell survival by blocking the niche defenses [7]. Its expression favors brain and lung metastasis of TNBC [44]. As previously reported, BrM2 cells have higher expression of the SerpinB2 protein compared to MDA-MB-231 cells [7,27,28]. Importantly, we found that SerpinB2 mRNA expression was promoted by the zinc cellular content. In BrM2 and MDA-MB-231-LM2 cells, the regulation was observed at the protein level as well. However, independently of the zinc condition studied, SerpinB2 mRNA expression levels in parental MDA-MB-231 cells did not reach BrM2 levels. As a consequence, SerpinB2 protein was not detected by Western blot in this cell line. This indicates that, besides zinc content, an additional transformative process must undergo in MDA-MB-231 parental cells during cancer progression to express sufficient SerpinB2 in order to escape from niche’s defenses against cancer cells’ invasion. In a more general view, SerpinB2 expression has been shown to be more frequently overexpressed in TNBC cells than in other breast cancer subtypes. It has been proven to be a poor prognostic factor linked to lymph node metastasis. miRNA-200c/141 has been shown to promote SerpinB2 expression specifically in TNBC cells [44]. The possible regulation of this microRNA by zinc is not established yet. However, there is a negative feedback loop between miRNA-200c/141 and the zinc-dependent transcription factor ZEB1 that deserves further study. 

In the metastatic process, cells often become dormant before generating a new tumor in the secondary niche [45]. Zinc homeostasis has been shown to modulate cell proliferation, and its dysregulation is involved in the growth of some cancer subtypes [29,37,42,43,46]. Interestingly, we showed that BrM2 cells proliferated slower than MDA-MB-231 cells. Moreover, in both cell lines, the proliferation rate was negatively affected by zinc, without an impact on cell survival. These results were unexpected, considering the roles recently described for Zip6 and Zip10 in the mitosis of MCF-7 cells [29]. The cancer subtypes and metabotropic action of these transporters could be behind this discrepancy. Besides, dormancy is a characteristic of CSCs [30]. This subpopulation is able to stay dormant for years in the secondary tumor and escape treatment, leading to relapses over time. In TNBC, cancer stem cells have been reported to be especially important for its classical progression [31].

Moreover, recently, SerpinB2 was proposed as a CSC tumorigenicity marker [32]. In this scenario, we found that zinc boosted CSCs’ potential to form new tumors. First, we found higher TSFE and CD44(+)CD24(−/low) populations in the BrM2 cells, with higher zinc content, than in the MDA-MB-231 line, indicating that there are greater CSC subpopulations within BrM2 cells and confirming their high metastatic potential. Moreover, we found that zinc induced tumorigenicity in the MDA-MB-231 line. In addition, we saw that the CD44(+)CD24(−/low) subpopulation was enriched in zinc, with CD44(+) as the specific marker associated with higher zinc content. In this context, it has been reported that ZEB1 regulates the CSC population of prostate cancer, also characterized by CD44 expression [35]. Interestingly, the presence of zinc reduced the ALDH marker in the tumorspheres of MDA-MB-231 and BrM2 cell lines. The enrichment of the CD44(+)CD24(−/low) subpopulation together with the reduction of ALDH suggest that zinc might displace breast CSCs towards a mesenchymal-like state [33]. Considering that mesenchymal-like CSCs are less proliferative than epithelial-like CSCs, the lower proliferation observed in our cells with increasing zinc concentrations is in agreement with this scenario. Finally, in our model of TNBC brain metastasis, we did not find zinc-dependent expression with either ZEB1 or the established drivers of CSC tumorigenesis, Nanog, SOX-2, and Oct-4 [34]. More research in this direction is needed. 

We wanted to understand the mechanistic processes driven by zinc that increase the metastatic potential in TNBC. Therefore, we modified MDA-MB-231 zinc homeostasis in order to resemble that observed in the BrM2 line. Zip4 is one of the proteins upregulated in BrM2 cells compared to the parental line. This transporter has been associated with cancer progression and the acquisition of prometastatic features in several types of cancer [36,37,38,46,47]. In our MDA-MB-231 cell model, Zip4 overexpression increased the cellular zinc content. However, that effect, similar to zinc supplementation conditions, was not sufficient to increase SerpinB2 protein expression. This result further indicates that zinc influx does not act as the main driver to acquire the brain metastatic phenotype observed in BrM2 cells and cancer patients [7] but promotes SerpinB2 expression once this phenotype is established. Nevertheless, we confirmed the tumorigenic properties of Zip4 expression previously described in other types of cancer. Thus, we observed an induction of tumorsphere formation in MDA-Zip4 cells. These results are in agreement with the reported role for Zip4 in CSCs of ovarian cancer [47]. In conclusion, Zip4 overexpression facilitated tumorigenicity in TNBC, but it was not enough to confer MDA-MB-231 cells with all of the metastatic potential and secondary niche adaptation observed in the BrM2 line.

## 4. Materials and Methods

### 4.1. Cell Culture

MDA-MB-231-BrM2 cells were kindly provided by Joan Massagué at Memorial Sloan Kettering Cancer Center, New York City, NY, USA. Cells were grown in DMEM supplemented with 10% FBS, 1% L-glutamine, and 1% penicillin and streptomycin at 37 °C in a humidified 5% CO_2_ atmosphere. When indicated, FBS was incubated according to the manufacturer’s instructions with Chelex 100 resin (Bio-Rad Laboratories, Hercules, CA, USA) to generate Zn^2+^-free growth medium. ZnSO_4_ was added as needed to the final medium to generate specific Zn^2+^ concentration conditions.

The MDA-MB-231 constitutively overexpressing the Zip4 cell line was generated using a pMSCVpuro-hZip4 plasmid and selected with 0.25 µg/mL of puromycin in the growth medium. The selection was removed to carry out the experiments.

### 4.2. Zinc Measurements

Cells were seeded and grown in 24-well plates until reaching 80% confluence. Cells were incubated with 25 µM of Zinquin (Sigma-Aldrich, Darmstadt, Germany) for 30 min at 37 °C (5% CO_2_) in an isotonic solution containing (in millimoles) 140 NaCl, 5 KCl, 1.2 CaCl₂, 0.5 MgCl₂, 5 glucose, and 10 Hepes (300 millimoles/liter, pH 7.4) plus different concentrations of zinc. Cells were then dissociated with Trypsin 0.05% in 0.53 mM EDTA and washed with PBS. Fluorescence was quantified using a LSRII flow cytometer. Further analysis was performed using Flowing Software (Turku Bioscience, Turku, Finland).

### 4.3. Cell Viability and Proliferation Assays

Cells were exposed to different concentrations of zinc in 24-well plates for 48 h, reaching 80% confluence. Then, MTT reagent was added to obtain a final concentration of 0.5 mg/mL. Cells were incubated for 2–3 h at 37 °C. After that, supernatant was removed, and cells were resuspended in 100 µL of DMSO. Absorbance was read at 590 nm.

In order to study cell proliferation, 5000 cells per well in 12-well plates were seeded. Cells were counted at 3, 5, and 7 days with a Neubauer chamber.

### 4.4. Real-Time RT PCR

Cells were seeded and grown in 6-well plates until reaching 80% confluence; we incubated the cells at the time and zinc concentration indicated. Total RNA from cells was extracted using a NucleoSpin RNA isolation kit (Macherey-Nagel, Düren, Germany). RNA was measured using a NanoDrop 1000 spectrophotometer (Thermo Scientific, Waltham, MA, USA). cDNA generation was conducted using a SuperScript Reverse Transcriptase system (Invitrogen, Waltham, MA, USA). Quantitative PCR was performed using SYBR Green (Applied Biosystems, Waltham, MA, USA) in the QuantStudio 12K system (Applied Biosystems, Waltham, MA, USA). Primers are listed in Table 1.

Relative mRNA abundance was calculated using the ΔΔCT method and plotted as indicated.

### 4.5. Western Blotting

Cells were seeded in 6-well plates; we incubated the cells at the time and zinc concentration indicated. Lysis was performed with 30 μL of lysis buffer containing 50 mM Tris-HCl at pH 7.4, 150 mM NaCl, 0.5% Nonidet P-40, and EDTA-free protease inhibition cocktail (Roche). Lysates were vortexed for 30 min at 4 °C and centrifuged at 10,000× *g* to remove aggregates. Lysates were boiled for 5 min at 95 °C and placed on ice for 1 min. Then, 20 μL of each sample were loaded onto a 12% polyacrylamide gel. After electrophoresis, proteins were transferred to nitrocellulose membranes using the iBlot system (Invitrogen, Waltham, MA, USA). Membranes were blocked with 5% milk in TBS-Tween 0.1% for 1 h at room temperature. Primary antibodies were diluted in blocking solution: anti-SERPINB2 (ab47742, Abcam) at 1:500, anti-Zip4 (20625-1-AP, Proteintech) at 1:500, and anti-GAPDH (ab8245, Abcam) at 1:1000 and incubated overnight at 4 °C. Anti-rabbit or anti-mouse HRP secondary antibodies (1:1000; GE Healthcare) were used. The ChemiDoc XRS+ system (Bio-Rad, Hercules, CA, USA) was used to obtain high-quality images. Quantity One Software (Bio-Rad) was used to analyze the results.

### 4.6. Tumorsphere Formation and Analysis

For this analysis, 10,000 MDA-MB-231 or BrM2 cells were plated onto 6-well ultra-low attachment plates (Corning). They were incubated for 21 days at 37 °C in a humidified 5% CO_2_ atmosphere with a serum-free medium (Gibco) containing B27 (Gibco), 20 ng/mL EGF (Sigma), 20 ng/mL bFGF (Gibco), and 4 g/mL heparin (Sigma) supplemented with zinc as needed. Then, 50 randomized pictures were taken per well using the Zeiss Cell Observer HS. Tumorspheres ≥100 µm that were formed in each replicate were counted with ImageJ image processing software (developed by NIH, Bethesda, DC, USA). The percentage of cells with the ability to form spheres, termed the tumorsphere formation efficiency (TSFE), was calculated as follows: (number of spheres that formed/number of single cells that were plated) × 100.

In order to analyze markers of stemness by flow cytometry, tumorspheres were centrifuged for 5 min at 300× *g* and washed twice with 1× PBS. Then, 110 uL of PBS were used to resuspend the cells. Cells were counted using a Newbauer chamber. Cells were stained with PE Mouse Anti-Human CD24 and APC Mouse Anti-Human CD44 antibodies (BD Bioscience), according to the manufacturer’s instructions. Cells were centrifuged for 5 min at 300× *g* and resuspended in 250 μL of PBS. Then, 1/1000 of DAPI (Thermo) was added. Results were obtained using a LSRII flow cytometer. To analyze the aldehyde dehydrogenase expression, the AldeRed ALDH Detection Assay Kit (Sigma-Aldrich) was used according to the manufacturer’s instructions. Results were obtained using a Fortessa flow cytometer. Further analysis was performed with Flowing Software (Turku Bioscience).

### 4.7. Migration Assay

For the assay, 5000 cells were seeded in a multi-well 6 plate and left to grow for 24 h. We took several pictures with a 10× objective in a Zeiss Cell Observer HS per well every 10 min for 24 h. We analyzed cell migration with FiJi software using the TrackMate Extension plug-in.

### 4.8. Invasion Assay

SPLInsert™ Hanging 24-well plates with a pore size of 8 μm (Lab Clinics) were used. The membrane was coated with 60 μL of Matrigel (Corning) diluted 1:3 in serum-free medium for 1 h at 37 °C. Then, 30,000 cells were seeded and left to grow and invade for 72 h. In each case, the medium of the upper chamber contained the indicated zinc concentration. The bottom chamber medium was the one used for normal cell culture. The media of the bottom chamber was re-collected, and 500 μL of trypsin were used to detach the cells in this chamber during 1 h at 37 °C. The trypsin was also re-collected, and we quantified the number of cells obtained from the bottom chamber with the LSRII flow cytometer.

### 4.9. Immunostaining

Cells were incubated in serum-free media with the primary antibody Zip4 (20625-1-AP, Proteintech, Rosemont, IL, USA) at 1:200 for 1 h at 37 °C. After being washed with PBS, cells were fixed with 4% PFA for 10 min at RT. Then, cells were incubated with blocking solution (1% PBS, 2% BSA) for 1 h at RT, followed by 1:1000 dilution of the secondary antibody in blocking solution. Coverslips were mounted with Fluoromont-G (SouthernBiotech, Birminghan, AL, USA). Pictures were taken with a Leica TCS-SP8 confocal microscope with a 63 × 1.40 immersion oil objective.

### 4.10. Statistics

All data are shown as mean ± SEM. In all cases, a D’Agostino–Pearson omnibus normality test was performed before any hypothesis contrast test. Statistical analysis and graphics were performed using GraphPad. For data that followed a normal distribution, we applied either Student’s *t*-test (when comparing MDA-MB-231 and BrM2 cells in basal conditions) and one-way analysis of variance (ANOVA) followed by Tukey’s post hoc test (when comparing one cell line in different zinc concentrations) or Dunnett’s multiple comparison (when comparing against control conditions). For data that did not fit a normal distribution, we used Mann–Whitney’s unpaired t-test and nonparametric ANOVA (Kruskal–Wallis) followed by Dunn’s post hoc test. The criterion for significant difference was a final value of *p* < 0.05.

## 5. Conclusions

Altogether, considering the different steps that a breast cancer tumoral cell must undergo to metastasize in the brain, our work highlights the importance of zinc homeostasis in two of the steps: SerpinB2 production and cancer stemness. The transcriptional pathways involved in this regulation remain unclear, and further research must be conducted to fully understand the scope of this transformative process in TNBC cells. Importantly, our work suggests that the use of zinc in breast cancer patients should be discouraged.

## Figures and Tables

**Table 1:** Primers used for RT-PCR studies.

Primers	Forward Sequence (5′→3′)	Reverse Sequence (3′→5′)
Zip1	GATTGGGGAAGACACTTGACTGCT	GAAAGAGGAAGGGGATTTGTTTGG
Zip2	CCCTTGTCCTCTTGCTGTCACTCT	AGCTCCCGTGGAAGAATTTCTAGG
Zip3	GTGGAGATATGAGGACCCCCTGTT	GATGAACTCAGCGCTAACCGATCT
Zip4	AGACTGAGCCCAGAGTTGAGGCTA	TGTCGCAGAGTGCTACGTAGAGGA
Zip5	GAGCAGGAGCAGAACCATTACCTG	CAATGAGTGGTCCAGCAACAGAAG
Zip6	CATAGCCATGAAGAACCAGCAATG	GAGAATCAAAGTGGGAGGGCTCTT
Zip7	ACTGAAGGAGGAGCAGTGGACAGT	AGGCCCTAATGCCAAAGTAACCAT
Zip8	CCTCGGATTGATTTTGACTCCACT	AGCAGGATTTGCATAGCATGTCAC
Zip9	GCCTAAAGAACTGGAAAGCCCACT	GTGTTTCACTTGCTTGGTGGTGTT
Zip10	TAGCCGTCTTCTGTCATGACTGC	TCATAGAGGGCAATCACCAGCATA
Zip11	TCTCCTAAGCATTTTGGTGGCCTA	TCTCTTCTTTCCACAGGGCTCACT
Zip12	CAACCACTCAAGAAGCCTCATCAA	AAGTACTGCCTGGTGAAAGCCAAG
Zip13	AAGAAGATCGGGCTCCTGACAAC	GAGAACAGCACCATTACCACGATG
Zip14	CATTTGGTTTCAACCCTCTGGAAG	TTTCAGCCAGTAGCAAGCACTCTG
SerpinB2	GTTCATGCAGCAGATCCAGA	CGCAGACTTCTCACCAAACA
Metallothionein-1	ATCTGCAAAGGGACGTCAGA	ACGGGTCAGGGTTGTACAAA
Metallothionein-2	TCCTGCAAGAAAAGCTGCTG	TCTTTACATCTGGGAGCGGG
Metallothionein-3	ACACACAGTCCTTGGCACAC	AAGTGCGAGGGATGCAAAT′.
Metallothionein-4	GTGTCTGCATGTCTGGAGGA	TCTGAGCCTCCTTTGCAGAT
ZEB1	GATGATGAATGCGAGTCAGATGC	ACAGCAGTGTCTTGTTGTTGT
ZEB1-AS1	CCGTGGGCACTGCTGAAT	CTGCTGGCAAGCGGAACT
Oct4	ACATCAAAGCTCTGCAGAAAGAACT	CTGAATACCTTCCCAAATAGAACCC
Sox2	AAATGGGAGGGGTGCAAAAGAGGAG	CAGCTGTCATTTGCTGTGGGTGATG
Nanog	ACATGCAACCTGAAGACGTGTG	CATGGAAACCAGAACACGTGG
GAPDH	GGAGTCCACTGGCGTCTTC	TGGCTCCCCCCTGCAAATG

## Data Availability

The data presented in this study are available on request from the corresponding author.

## Supplementary Materials

The following are available online at https://www.mdpi.com/article/10.3390/ijms22179188/s1.
