# Supplementary material for: Zinc Favors Triple-Negative Breast Cancer’s Microenvironment Modulation and Cell Plasticity
Source: Int J Mol Sci. 2021 Aug 25;22(17):9188. doi: 10.3390/ijms22179188 (PMC8431059; doi:10.3390/ijms22179188)
Supplement: Supplementary file 1 [file ijms-22-09188-s001.zip › ijms-1318707-supplementary.pdf]

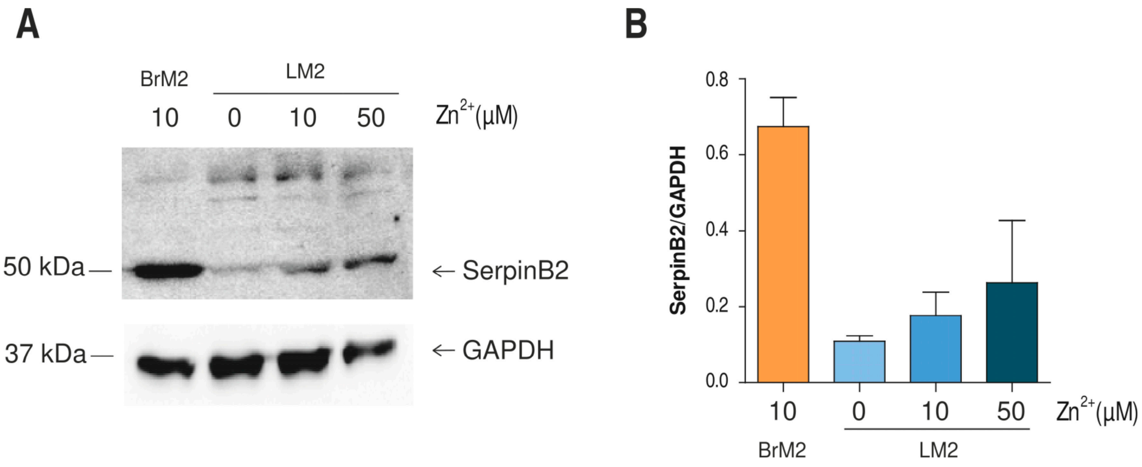

Supplementary Figure S1. SerpinB2 expression in MDA-MB-231-LM2 cells. (**A**) Representative Western blot against SerpinB2 and GAPDH in BrM2 and LM2 after 24 h of treatment with 0, 10, and 50 μM of $ZnSO_4$. (**B**) Quantification of SerpinB2 protein expression normalized by GAPDH protein expression in BrM2 and LM2 cells after 24 h of treatment with 0, 10, and 50 μM of $ZnSO_4$ (n = 2).

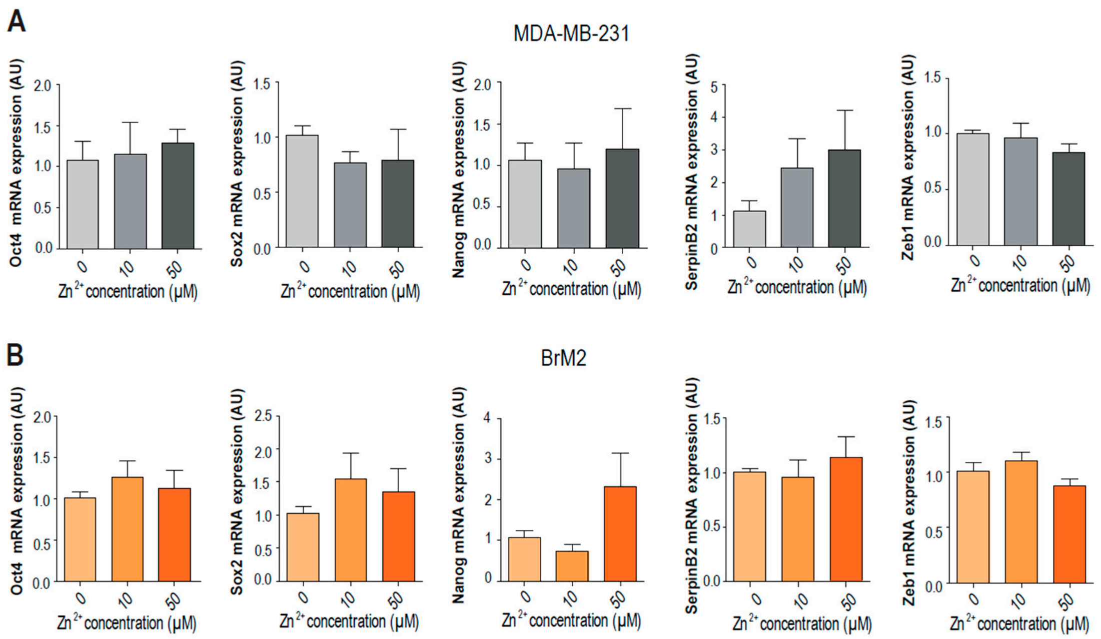

Supplementary Figure S2. RNA expression levels of stem-cell markers (**A**). Real-Time RT PCRs comparing the MDA-MB-231 tumorspheres expression of Oct4, Sox2, Nanog, SerpinB2 and Zeb1 in 0, 10 and 50 μM of $ZnSO_4$. 2^{-DDCT} plotted using GAPDH as housekeeping gene and the 0 μM of $ZnSO_4$ condition as the control. (n = 4) (**B**) Real-Time RT PCRs comparing the BrM2 tumorspheres expression of Oct4, Sox2, Nanog, SerpinB2 and Zeb1 in 0, 10 and 50 μM of $ZnSO_4$. 2^{-DDCT} plotted using GAPDH as housekeeping gene and the 0 μM of $ZnSO_4$ condition as the control. (n = 3–8).

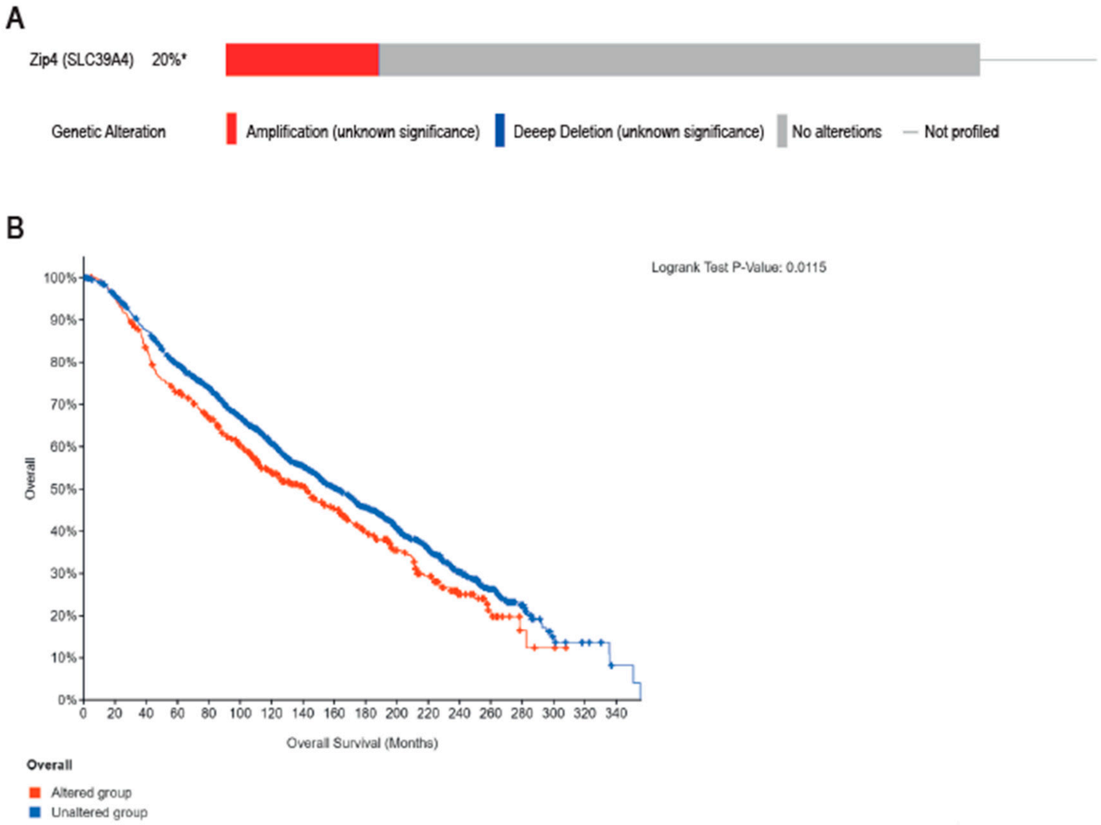

	Number of Cases, Total	Number of Events	Median Months Overall (95% CI)
Altered group	**401**	**247**	**143.00 (116.43 - 163.20)**
Unaltered group	**1577**	**897**	**161.67 (150.60 - 173.03)**

Supplementary Figure S3. Zip4 characterization in breast cancer patients. Extracted from cBioPortal for cancer genomics (METABRIC, Nature 2012 & Nat Commun 2016).
